# Collecting syndromic surveillance data by mobile phone in rural India: implementation and feasibility

**DOI:** 10.3402/gha.v8.26608

**Published:** 2015-04-02

**Authors:** Vishal Diwan, Deepak Agnihotri, Anette Hulth

**Affiliations:** 1 Department of Public Health and Environment, R.D. Gardi Medical College, Ujjain, India; 2 International Center for Health Research, R.D. Gardi Medical College, Ujjain, India; 3 Department of Public Health Sciences, Karolinska Institutet, Stockholm, Sweden

**Keywords:** syndromic surveillance, India, infections, informal providers, mobile technology

## Abstract

**Background:**

Infectious disease surveillance has long been a challenge for countries like India, where 75% of the health care services are private and consist of both formal and informal health care providers. Infectious disease surveillance data are regularly collected from governmental and qualified private facilities, but not from the informal sector. This study describes a mobile-based syndromic surveillance system and its application in a resource-limited setting, collecting data on patients’ symptoms from formal and informal health care providers.

**Design:**

The study includes three formal and six informal health care providers from two districts of Madhya Pradesh, India. Data collectors were posted in the clinics during the providers’ working hours and entered patient information and infectious disease symptoms on the mobile-based syndromic surveillance system.

**Results:**

Information on 20,424 patients was collected in the mobile-based surveillance system. The five most common (overlapping) symptoms were fever (48%), cough (38%), body ache (38%), headache (37%), and runny nose (22%). During the same time period, the government's disease surveillance program reported around 22,000 fever cases in one district as a whole. Our data – from a very small fraction of all health care providers – thus highlight an enormous underreporting in the official surveillance data, which we estimate here to capture less than 1% of the fever cases. Additionally, we found that patients from more than 600 villages visited the nine providers included in our study.

**Conclusions:**

The study demonstrated that a mobile-based system can be used for disease surveillance from formal and informal providers in resource-limited settings. People who have not used smartphones or even computers previously can, in a short timeframe, be trained to fill out surveillance forms and submit them from the device. Technology, including network connections, works sufficiently for disease surveillance applications in rural parts of India. The data collected may be used to better understand the health-seeking behaviour of those visiting informal providers, as they do not report through any official channels. We also show that the underreporting to the government can be enormous.

There are three main reasons why infectious disease surveillance data should be collected: monitoring its incidence so that authorities can be informed and take adequate preventive measures if necessary; assisting the health care sector in planning; and collecting baseline data so that interventions that have been implemented for a particular disease or situation may be evaluated.

India has a nationwide program, known as the Integrated Disease Surveillance Project (IDSP) (1), for the weekly reporting of surveillance data on epidemic-prone diseases to the authorities. Data are reported from sub-centres, primary health centres, community health centres, government and private hospitals, and medical colleges throughout the country. Both syndromic data and laboratory-verified cases are reported using standard case definitions (1). The syndromic data are reported on the document known as Form S. Six syndromes are included on that form (fever, cough, loose watery stool, jaundice cases, acute flaccid paralysis cases among those younger than 15 years, and other unusual symptoms leading to death or hospitalisation). In 2010, approximately 850,000 sub-centres in India sent weekly reports on syndromes (2).

Not all health care providers comply with the requirement of reporting suspected and confirmed cases, especially in the private sector (3), which is a potentially serious concern as 75% of all providers are found in the private sector (4). A second gap in reporting involves informal health care providers, which lack state-accredited medical qualification and are not authorized to practice allopathic medicine (5). The informal providers often lack regular medical education but may have at least some medical training. These individuals nevertheless examine patients and prescribe medication just like a qualified health care provider. Informal providers represent a large portion of the health care system in resource-limited areas of the world. A mapping of all health care providers in the state of Madhya Pradesh (a central state in India with nearly 80 million inhabitants) in 2004 found a total of 207,916 health care providers, excluding traditional birth attendants in the state as a whole, of which 89,090 (43%) were unqualified (6). Individuals living in rural areas are more likely to see an informal provider compared to urban populations (7), partly because of the fact that formal providers are more likely to work in the urban areas (8). As the informal providers by definition are unregistered, they do not submit any data to the authorities through IDSP or other channels.

A study conducted in 2011 in Maharashtra, another Indian state, identified a number of additional challenges for IDSP (9). One conclusion was that improving the quality of surveillance should be a high priority for the government. The same study showed that 76% of the districts surveyed relied on Form S for outbreak detection. In order to find outbreaks at the village level, Form S data to be collected from the sub-centre level (10).

Low technology applications are being used more and more frequently in resource-limited settings to complement conventional surveillance (11). For example, in Papua New Guinea, a mobile phone-based syndromic surveillance system was shown to provide more timely, complete, and sensitive surveillance data (12). It has also been suggested that automation of data transmission will improve data quality and the timeliness of data submitted through the IDSP in India (13).

In the present study we describe a qualitative evaluation of a mobile phone-based syndromic surveillance platform for submitting reports on symptoms of patients seeking health care in rural India. The data collected provide richer knowledge about the demographics and symptoms of individuals seeking care; the data are presented in this paper, with a particular focus on those visiting informal providers.

## Methods

### Study setting

This cross-sectional study was conducted in two districts of Madhya Pradesh, India (districts are independent administrative units within a state). Madhya Pradesh is one of the largest states in India, covering an area of 308,000 square kilometres with nearly 80 million inhabitants (14). Its health indicators are among the poorest figures in the country (15). The two districts studied are situated in western Madhya Pradesh; each has close to 2 million inhabitants and the literacy rate is above 70%. The majority of the population in the study districts are engaged in agriculture and related fields for their livelihoods. The health care providers were selected based on their willingness to cooperate in the mobile-based surveillance study. Within the two districts, a total of nine providers were included. Two of them were from private charitable hospitals, one from a government community health centre, and six were informal health care providers who work in rural parts of the districts.

### Technical framework

In order to establish if the reporting of infectious disease symptoms could be supported by mobile phones, we used a technical framework developed for reporting data indicative of infectious diseases (16). The purpose of that EU-funded project was to investigate if syndromic surveillance data could be collected from rural sites in China; data on sales of medication, school absenteeism, and visits to primary health care units were collected and analysed. In that project, data were entered from a computer into a designated platform which was accessible through the Internet (17). For the purpose of our study, this platform was extended to support reporting from mobile devices. An implementation of the extended platform was made on a designated server in India, to which all collected data were submitted. Six data collectors were hired, each equipped with a smartphone for data submission.

The first version of the implementation supporting mobile devices required Internet access for the data collection, as the forms were stored on the server. Since the data collectors experienced problems with the network when the study was launched, the ability to store forms in offline mode and submit them later when connected was implemented. In this mode, the forms could be saved on the mobile device and automatically sent by bulk when there was a reliable network connection.

### Data collection and submission

The data collectors were posted in the providers’ clinics during working hours. They worked closely with the providers and collected information on demographics and symptoms of the patients. Information on nine symptoms was collected, defined as fever, cough, sore throat, runny nose, diarrhoea, vomiting, abdominal pain, body ache, and headache. A tenth and final field dubbed other was used for any reason that did not fit into one of those categories. Patients who presented with more than one symptom had each symptom recorded separately.

The data were entered on a paper form in consultation with the patient, then entered on an electronic form on the smartphone resembling the paper form and submitted to the central server. Software-related issues such as duplicate data entry were monitored regularly and duplicates removed from the dataset. The place of residence of the patients was geocoded and added to each entry in the dataset.

Data were collected from 1 January 2013 to 30 June 2013, 6 days per week. The data collector had 1 day off per week; the specific day was the same weekday for each individual varied among the data collectors as a group. Before primary data collection, a pilot study was conducted for approximately 1 month to check the data collection instruments, mobile networks, and other logistical factors.

### Evaluation of technical framework

The evaluation of the implementation and the feasibility of the surveillance system was carried out in a qualitative manner. We evaluated the usability of the framework by reviewing how easy it was to use the device and the software. We also evaluated both stability and acceptability (18). We evaluated the former through a questionnaire in which, for two discontinuous weeks, the data collectors were asked to self-report irregularities with the mobile daily; they reported for the 6 days of their work week, did not report for the week following, then reported again for their next six workdays. We also queried the number of times that they had to recharge the device battery and their perceptions of the quality of the Internet connection.

A focus group discussion was performed with the data collectors using a structured interview guide. All interviews were conducted in Hindi and subsequently translated to English to allow for a collaborative analysis of the results. The results of the focus group discussions formed the basis for evaluating the acceptability and usability of both the software and mobile device.

### Data analysis

The data were transferred from the server to Excel spreadsheets and thereafter to PASW Statistics 18.0 for cleaning and quality checks. In this paper, we present basic descriptive statistics drawn from the collected data with respect to symptoms, age, and sex. The analyses are presented with respect to whether the provider is governmental, formal (private), or informal.

We obtained data submitted to the IDSP for one of the two districts from that district's IDSP office. We compared the data that we collected from the clinics in that district to data reported through the IDSP for the same time period (January 1 to June 30, 2013) for symptoms reported in both systems.

### Permissions

The Ethics Committee of R.D. Gardi Medical College, Ujjain approved the study. Permission to conduct the study was also granted from the IDSP district office in Ujjain.

## Results

### Evaluation of technical framework

The questionnaire on battery charging and connectivity was completed for seven of the nine study sites. All data collectors participated in the structured focus group discussion.

#### Stability

The data collectors experienced good network connections on 42% of the days during the 2 weeks that battery charging and connectivity were studied. During those weeks the data collectors worked on average 4 h and 45 min per day. However, even during days when they deemed the connection to be good, they still experienced network problems on 18% of the days. As one collector reported: *Once or twice a day we used to have problems with the Internet connection*.

There were also problems with electricity on 7% of the days. Overall, the seven sites reported 26% of days had good connections. The data collector at the urban site for which the questionnaire was completed experienced good connectivity on 60% of the days during the period analysed. Even at this site, though, there were frequent network problems during the days with good network connection, to the same extent as in the rural sites.

The battery was, with few exceptions, charged every day. There were three instances reported in which the mobile phone ran out of charge during working hours and could not be used for further submission of data. Furthermore, one of the health workers stated in the focus group discussion that the mobile often ran out of charge during a full day:

Initially it was good but slowly it started losing charge very quickly. We had to charge our battery after two hours in the morning and again after two hours in the evening.

No other irregularities were reported during the 2 weeks that data collectors were asked to fill out the questionnaire.

#### Acceptability

All health care providers agreed to the data collectors’ sitting with them and noting their patients’ symptoms, although some were initially quite reluctant:

They must have wondered what we would do with this information. They must have been scared that we would start some new project that might take away their patients. The private doctors were scared but government doctors didn't have this problem.

Sometimes the patients did not want the data collector to be present while they saw the provider, in which case the provider reported the symptoms to the data collector afterwards. Some data collectors wanted to collect more information than requested and also saw the need for diagnosis-specific data collection:

I had no problem in filling out the forms, but the only issue was that the questions were not adequate; for example, there was no information on malaria, tuberculosis, or other major diseases. So, I wrote that in on my own in the bottom of the form. Different people were from different places and there were not full addresses and if possible there should be provision for people to put their mobile numbers as well.

The data collectors also were asked to perform tasks beyond collecting data:

Some doctors thought that they had an employee to do work like entering the OPD register, giving medicines, etc. We cooperated with them by doing their odd jobs.

#### Usability

Five of the six data collectors used a smartphone for the first time during this study. All data collectors felt comfortable with entering the data on the smartphone within a week. They stated, however, that they would have preferred more training in using the device. The offline mode that was added very early in the study was appreciated by the data collectors:

In offline mode we can do our work without an internet connection and in online mode we have to connect again and again to a network. Offline mode saves time and more forms are completed over a given period.

The data collectors found the screen a bit too small for the form they had to fill in, which led to mistakes in entering the data. The data collectors were also concerned that they had no feedback on the data they had collected and that they could not see the number of patients with any one symptom. The health care providers also requested this information, according to the data collectors.

### Results of analysis of collected data

A total of 21,326 entries were executed on the mobile-based disease surveillance system during the study period. When checked for quality, 902 were found to be either previously undetected duplicate entries or incomplete entries. These were removed from the final dataset, which thus consisted of 20,424 patient encounters. These patients are described in more detail in Table 1. Eleven percent of those seeing any health care provider in our study were 5 years old or younger. Eighty-three percent of all patients reported in the mobile-based systems visited a private provider, whether informal or formal. Twenty-one percent of females visited the governmental provider compared to only 14% of males (these data not presented in the table). Twenty-nine percent of patients visiting the governmental provider were 5 years old or younger. The patients travel- led on average 29 km to the health care providers for consultation, from more than 600 villages.

**Table 1:** Numbers of patients for which information was collected from the nine health care providers, grouped by governmental, private (formal), and informal

	Total number of patients	Up to 5 years old (%) out of patients reported for each provider	% Male/female	No. of residential villages of visiting patients	Median distance km (max, min)
Government	3,465	18.41	43.3/56.7	222	24 (87, 0)
Formal 1	2,553	4.2	44.4/55.6	245	35 (76, 0)
Formal 2	2,503	5.3	45.6/54.4	19	49 (7, 0)
Informal 1	3,862	10.8	52.9/47.1	394	27 (90, 0)
Informal 2	925	3.5	82.7/7.3	19	27 (100, 0)
Informal 3	1,873	9.8	64.8/35.2	54	29 (93, 0)
Informal 4	2,182	13.4	57.4/42.6	68	13 (92, 0)
Informal 5	2,168	15.5	60.1/39.9	74	12 (92, 0)
Informal 6	893	4.5	80.6/19.4	82	42 (112, 0)
Total	20,424	10.7	45.8/54.2	602	27

**Fig. 1:**
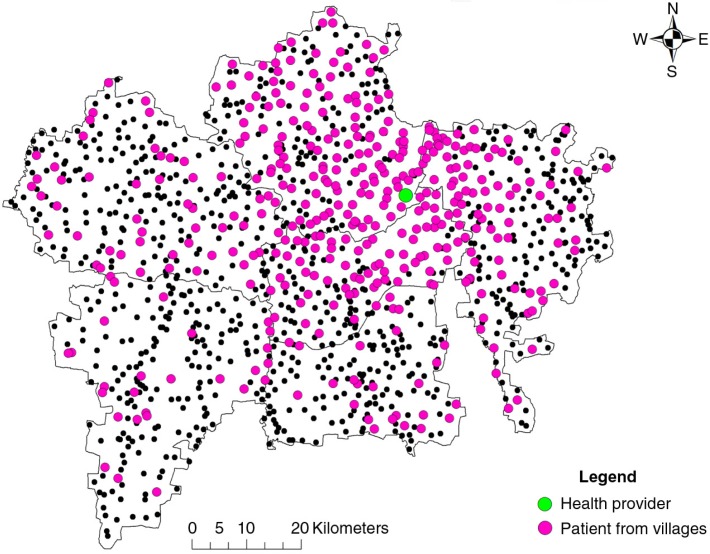
The residential village (pink dot) of all patients visiting one of the informal health care providers (green dot) during the entire study period.

Figure 1 is a map of all residential villages of the patients visiting one informal health care provider in the entire study period. Table 2 shows that informal providers attract patients from a large number of villages. The pattern looks very similar for the other informal providers, while for the formal providers, including the governmental facility, the villages from which the patients travelled are closer as a whole to the provider.

**Table 2:** Numbers of patients presenting with various, overlapping symptoms per health care provider

Type	Fever	Cough	Body ache	Headache	Runny nose	Vomiting	Abdominal pain	Diarrhoea	Sore throat
Government	1,706 (49.2)	1,301 (16.6)	2,093 (60.4)	1,502 (26.1)	1,194 (27.0)	354 (37.7)	589 (14.2)	195 (10.0)	132 (9.4)
Formal 1	523 (20.5)	545 (7.0)	462 (32.7)	2,045 (6.7)	332 (7.5)	263 (6.2)	487 (11.7)	116 (6.0)	80 (5.7)
Formal 2	403 (16.1)	353 (4.5)	77 (18.5)	2,067 (5.8)	237 (5.4)	281 (6.6)	458 (11.0)	298 (15.3)	316 (22.5)
Informal 1	3,562 (92.2)	2,300 (29.4)	2,338 (60.5)	1,442 (32.1)	744 (16.8)	1,596 (37.7)	657 (15.8)	239 (12.3)	220 (15.7)
Informal 2	191 (20.6)	487 (6.2)	234 (25.3)	683 (3.2)	165 (3.7)	119 (2.8)	206 (5.0)	41 (2.1)	102 (7.3)
Informal 3	1,170 (62.5)	682 (12.6)	760 (40.6)	1,485 (5.2)	488 (11.0)	566 (13.4)	545 (13.1)	357 (14.6)	347 (24.7)
Informal 4	1,156 (53)	682 (8.7)	416 (19.1)	1,401 (10.4)	538 (12.2)	497 (11.7)	487 (11.7)	357 (18.4)	54 (3.8)
Informal 5	923 (42.6)	700 (8.9)	145 (19.3)	1,565 (8.0)	542 (12.3)	436 (10.3)	504 (12.1)	363 (18.7)	65 (4.6)
Informal 6	136 (15.2)	475 (6.1)	835 (16.2)	704 (2.5)	184 (4.2)	123 (2.9)	226 (5.4)	49 (2.5)	87 (6.2)
Total	9,770 (47.8)	7,833 (38.4)	7,701 (37.7)	7,530 (36.9)	4,424 (21.7)	4,235 (20.7)	4,159 (20.4)	1,942 (9.5)	1,403 (6.9)

For each provider we also present the percentage of patients with that symptom out of all patients reported for the health care provider in question.

Table 2 describes in detail the collected symptoms per provider: 48% of the patients presented with fever, 39% with cough, 38% with body ache, 37% with headache, 22% with runny nose, 21% with vomiting, 21% with abdominal pain, 10% with diarrhoea, and 7% with sore throat.

The number of fever cases reported to the IDSP for the district was 22,556 for the study period, while for the eight providers included in our study and located in the same district, 9,770 cases with fever were reported. For cough the number reported to IDSP was 3,463 and for diarrhoea 6,120. The health care providers in the district reporting in our study saw 7,833 cases with cough and 1,942 cases with diarrhoea. The district is understood to have approximately 2,941 health care providers (19), which means that the reporting to the IDSP catches about 0.6% of the actual number of cases of fever and that does not take into account that the data collectors only worked part of the day and thus did not report all the cases that provider saw. For cough the corresponding figures are 0.1% and for diarrhoea 0.08%.

## Discussion

To our knowledge this was the first report where informal providers in India were involved in syndromic disease surveillance. The evaluation showed that the technical infrastructure that was used for collecting syndromic surveillance data in a rural part of central India functioned sufficiently well. There were issues with network connectivity and with the batteries, but overall the system can be deemed adequate and was accepted by the data collectors. There is a potential risk that the data collectors were more positive during the focus group discussion because they felt loyalty to the project and also did not want to risk of not getting other assignments in the future by expressing negative views. Some of the data collectors would have preferred more training, but no evidence of insufficient training showed in their handling of the system itself. One of several challenges in using information technology for public health matters is the lack of computer literacy among health professionals (20). In our study, the data collectors needed only a couple of hours of training to be able to handle a smartphone for entering and submitting the surveillance data, although they had never used a smartphone or even a computer. However, there could have been more training in how the symptoms should be classified based on some form of symptom definitions; that would have assured that the data collectors had a more harmonised way of selecting the symptoms. Additionally, as some of the data collectors wanted diagnosis-specific fields in the form, more time should have been spent on explaining the surveillance scheme, what it was designed to track, and what it explicitly did not seek, such as detailed personal contact information.

The data collectors wanted more feedback on the data they submitted, as did the health care providers themselves. If these data were compiled and reported back to the providers, they would serve as the only numerically descriptive measures on their caseloads, as they rarely keep any records on the patients they see.

In the present study, we also collected data on symptoms from informal providers. As the informal providers constitute a large part of the health care sector in India, particularly in the rural areas, there is a significant amount of information that is never collected by IDSP. In a recent review on informal health care providers in various parts of the world – where 195 papers containing any kind of recommendation on how to support informal providers better and thus the care-seeking population were included – the three categories most often mentioned were educational interventions (*n*=66), regulations and enforcement (*n*=61) and collaboration and engagement (*n*=38) (7). It is difficult, though, to see that statistics from informal providers would be accepted by the authorities, as that would require them to acknowledge this sector. It could also very well be that the informal providers would not want to report, as they may lack trust in the authorities.

The kind of data we collected could be useful in two ways, even if not included in any official reporting. First, they can be used to estimate the size of the underreporting in a system like IDSP. Second, they can be used to better understand the care-seeking behaviour of those visiting informal providers. In the analyses presented we have given some examples of what can be culled from such data. However, as the number of clinics is small, we must be careful in drawing any broad, firm conclusions from these data. They should be seen more as giving indications on where to focus future research.

Examples of areas that are fruitful for further study are the differences in symptom pattern between the IDSP and the health care providers in our study, and the fact that men tend to visit private providers, while women see informal providers and the children primarily visit the governmental health centre. It has been shown previously that one reason why patients visit an informal provider is the proximity to the provider (21). However, we see in our results that many patients do travel significant distances to see a health care provider, whether formal or informal. Some of those persons can be assumed to become ill while visiting the place from which data are sent (but will be recorded with a residential address far from the provider). Because of the large number of persons who have travelled substantial distances, however, this can explain only a fraction of all those travelling.

In syndromic surveillance, it is often presupposed that the data used for surveillance are readily available in electronic format because they already have been collected for purposes other than disease surveillance (22). However, in many settings, such as rural areas in resource-limited parts of the world, this is far from the case. When surveillance data must be collected manually, higher costs result. Accessibility, visualisation, and cost related to data collection differ significantly among different available mobile tools (23). In the present study, we provided the data collectors with inexpensive smartphones. However, after only a couple of months they began to lose their charge several times a day. There may thus be costs that reveal themselves only in the medium or long terms.

There are few studies in the literature that have proven that mobile phones can improve surveillance beyond the pilot stage (24). From a technological point of view, there are very few challenges to scaling up the system. In our study we had designated data collectors who submitted the data. Should the system be scaled up, it would require the health care providers themselves to do the reporting. The challenge would thus be to motivate the providers to submit the data: ‘Perhaps most importantly, investing in human resources – through training in epidemiology, data management and analysis, use of computers, and other public health skills – would help address an important threat to public health security in developing countries’ (25).

## Conclusions

We presented a study in which we extended a technological platform for collecting syndromic surveillance data to support reporting from mobile devices. The platform was used to collect data from health care providers in a rural part of India. From the evaluation it became clear that even people who had not used smartphones or computers before could be trained quickly to fill out surveillance forms and submit them from the device. We can also conclude that the technology, including the network connection, works sufficiently well for these kinds of applications also in rural parts of a country like India. It is necessary, however, that the application be able to store data without network connection, as temporary network problems are frequent. Data on symptoms were collected from both formal and informal health care providers. These kinds of data may be used to better understand the health-seeking behaviour of those visiting informal providers in particular, as they do not report through any official channels. We showed that many patients travel far to see even informal providers. We also made a rough estimation of the underreporting of cases with notifiable symptoms to the IDSP, which is nothing less than enormous.

Collection of data is one end of surveillance; the other is the dissemination of the analysed information to relevant stakeholders, who in turn are those who must take action.
